# Fat Intake Is Not Linked to Prostate Cancer: A Systematic Review and Dose-Response Meta-Analysis

**DOI:** 10.1371/journal.pone.0131747

**Published:** 2015-07-17

**Authors:** Chang Xu, Fang-Fang Han, Xian-Tao Zeng, Tong-Zu Liu, Shen Li, Zheng-Yan Gao

**Affiliations:** 1 Department of Urology, Zhongnan Hospital of Wuhan University, Wuhan, China; 2 Center for Evidence-Based Medicine and Translational Medicine, Zhongnan Hospital of Wuhan University, Wuhan, China; 3 Department of Ophthalmology, Zhongnan Hospital of Wuhan University, Wuhan, China; Hunter College, UNITED STATES

## Abstract

**Background:**

Since the late 1960s, the average global supply of fat has increased by 20 g per capita per day. While fat intake has been considered a potential risk factor for prostate cancer (Pca), the hypothesis from previous epidemiologic studies remained equivocal.

**Materials and Methods:**

Relevant cohort studies were identified through a literature search in PubMed, ScienceDirect and Wiley Online Library up to March 1, 2015. A systematic review and dose-response meta-analysis were used to assess the relationship between fat intake and the risk for Pca.

**Results:**

We identified 14 cohort studies, which included 37,349 cases and a total of 751,030 participants. We found no evidence of a non-linear association between fat intake and the risk for Pca. Overall, the summarized relative risks for every 28.35 g increment a day was 0.99 (95%CI: 0.98, 1.01; P=0.94; n=13) for total fat intake, 1.00 (95%CI: 1.00, 1.00; P=0.72; n=9) for saturated fat, 0.99 (95%CI: 0.95, 1.03; P=0.55; n=7) for polyunsaturated fat, and 1.00 (95%CI: 0.95, 1.04; P=0.85; n=8) for monounsaturated fat. Additionally, there was no link to the risk for advanced stage Pca regarding total fat intake (RR=1.02, 95%CI: 0.96, 1.08; P=0.63; n=5), saturated fat (RR=0.96, 95%CI: 0.84, 1.11; P=0.61; n=6), polyunsaturated fat (RR=0.96, 95%CI: 0.79, 1.17; P=0.68; n=6), or monounsaturated fat (RR=0.96, 95%CI: 0.86, 1.07; P=0.42; n=6). Subgroup and sensitively analyses showed consistent results.

**Conclusion:**

Little evidence from published cohort studies supports the statement that total fat, saturated fat or unsaturated fat intake increases the risk for Pca or advanced stage Pca.

## Introduction

Prostate cancer (Pca) is the second leading cause of cancer death among American males [1] and has a crude incidence of 38.2 per 100, 000 men for 1-year prevalence and 151.2 for 5-year prevalence worldwide [2]. According to the National Cancer Control Institute (NCCN), an estimated 233,000 men were diagnosed with Pca in 2014, which accounted for 27% of newly diagnosed cancer cases [3].

The World Health Organization reports that since the late 1960s, the average global supply of fat has increased by 20 g per capita per day [4]. In many countries, such as America, Canada, Australia, France, Finland, New Zealand the incidence of prostate cancer has increased [2]. Previous epidemiology studies have reported potential correlations between fat intake and the risk for Pca [5, 6]. The mechanism is complex and unclear, one possible explanation may be the oxidative stress generated during fat metabolism [7, 8]. Other proposed mechanisms, including serum testosterone level [9], free radicals [10], and insulin-like growth factor levels [11] related to fat intake. Nevertheless, the relationship between fat intake and risk of prostate cancer remains controversial.

In several previous reviews and meta-analyses [12, 13, 14], total fat consumption was associated with Pca risk while saturated and unsaturated fat consumption were not. In another meta-analysis, however, no association was confirmed between fat intake and risk for Pca [15]. The current data on Pca risk and fat consumption were highly heterogeneous and insufficient. The limited study numbers or less robust design also made them low reliability. Therefore, we conducted a systematic review and dose-response meta-analysis, with more available cohorts and flexible design. We aimed to investigate the relationship between the consumption of different types of fats and the risk for Pca.

## Methods

We conducted our meta-analysis following the preferred reporting method for systematic reviews and meta-analyses (PRISMA) statement [16] (S1 PRISMA Checklist).

### Search Strategy

Eligible cohort studies were identified by searching PubMed, ScienceDirect, and Willey Online Library published up to March 1, 2015. Two reviewers (T.Z. Liu and Z.Y. Gao) independently searched each database and any disagreements were resolved by a methodologist (X.T. Zeng) for a final decision. A Kappa statistical test was used for measuring agreement [17]. The following search terms were used: “fat intake” OR “high-fat diet” OR “dietary fat” AND “prostate cancer” OR “prostate tumor” OR “prostate neoplasm” OR “prostate carcinoma” OR “prostate tumour”. References in identified articles were also reviewed. There was no language limit.

### Eligibility criteria

Because case-control studies may introduce considerable bias, particularly recall bias, only cohort or case-cohort studies were included in our meta-analysis [17, 18, 19]. The primary outcome of interest was any stage of Pca and the exposures analysed were total fat, saturated fat, or unsaturated fat intake. Secondary tumors from other organs were not considered. The estimated effect was either provided in the study or could be calculated from raw data. All studies included at least three quantitative categories of fat intake. Studies reporting animal fat (except for fish oil) were categorized as saturated fat. We found that most studies pooled vegetable and fish oils into total, saturated or unsaturated fat. Thus, vegetable and fish oils were not considered in this meta-analysis. Grey literature, meeting paper, and animal studies were excluded from this meta-analysis.

### Data extraction

From all included studies two experienced reviewers (S. Li and F.F. Han) extracted the first author’s name, publication year, country, study type, follow-up, age distribution, types of fat number of cases or person-years, serving size, adjusted or crude relative risk (RR) with 95% confidence intervals (CI), adjusted variables, and the degree of Pca using a standardized data collection sheet. When different models were used to adjust for confounders, we extracted the RR that controlled for the most factors. A third investigator (Z.Y. Gao) checked the data and corrected potential errors.

### Data conversion

For studies that measured fat intake with energy percentage, the data was converted to grams by multiplying by the mean daily total energy intake and then dividing by 9 (1 gram of total fat provides 9 Kcal energy). If studies did not report the mean daily energy intake, we assumed it to be 2,418 Kcal, which is the age–specific energy value for 50–71 year old males according to the National Cancer Institute [20]. This data conversion may overestimate the amount of saturated fat consumed and underestimate unsaturated fat consumption since saturated fat contains more calories than unsaturated fat.

### Statistics analyses

Relative risk (RR) was used to measure the risk. Odds ratios (OR) and hazard ratios (HR) were roughly regarded as relative risk (RR) [21]. Missing data was evaluated as described by Bekkering et al [22]. Briefly, if the number of non-cases was missing, the group sizes were assumed to be approximately equal. If the number of cases were missing, the reported RRs and non-control numbers were used to calculate the number of cases. There was no valid way to evaluate if the serving size was missing.

The dose-response meta-analysis was conducted in two steps. First, the generalized least-squares method estimated the coefficient per unit increment of exposure within each study. Second, the regression coefficients were combined in a random-effect model with the weight calculated by inverse variance [23, 24]. All effect sizes were logarithm transformed for the meta-analysis. The lowest exposure level served as the reference category in each study and the estimated log relative risk in the reference category was set to zero (log 1) [24]. Every 28.35 g (approximately 1 ounce) increment of fat intake per day was used to measure the dose-response relationship.

We used the mean value of the lower and upper boundaries of each category as the assigned dose. For open-ended lower categories, the assigned dose was calculated by dividing the cut-off point by 1.2. For open-ended upper categories, the cut-off point was multiplied by 1.2 [25]. The non-linear trends between total fat, saturated fat, and unsaturated fat intake and the risk for Pca were fitted by modelling both tails (left-tail and right-tail) restricted cubic splines with three knots at fixed 10th, 50th, and 90th percentiles of exposure distribution [26]. The Wald test was used to evaluate linearity or non-linearity trends by assuming the regression coefficient of the second spline equalled zero [26]. Some studies reported RRs by subtypes (such as sex or area), in our meta-analysis we pooled the subtypes using a fixed-effect model before including them in the overall analysis [19, 27].

The Egger’s test was used to determine publication bias, the I^2^ statistic assessed heterogeneity, and subgroup and sensitivity analyses evaluated whether the results were consistent. A random-effects meta-regression was used to assess which covariates in the subgroup analysis influenced the intervention effect [17]. All the analyses were performed using the Stata SE12.0 software (Stata SE 12.0 Corp LP, College Station, Texas, USA).

## Results

### Search results

**Fig 1:**
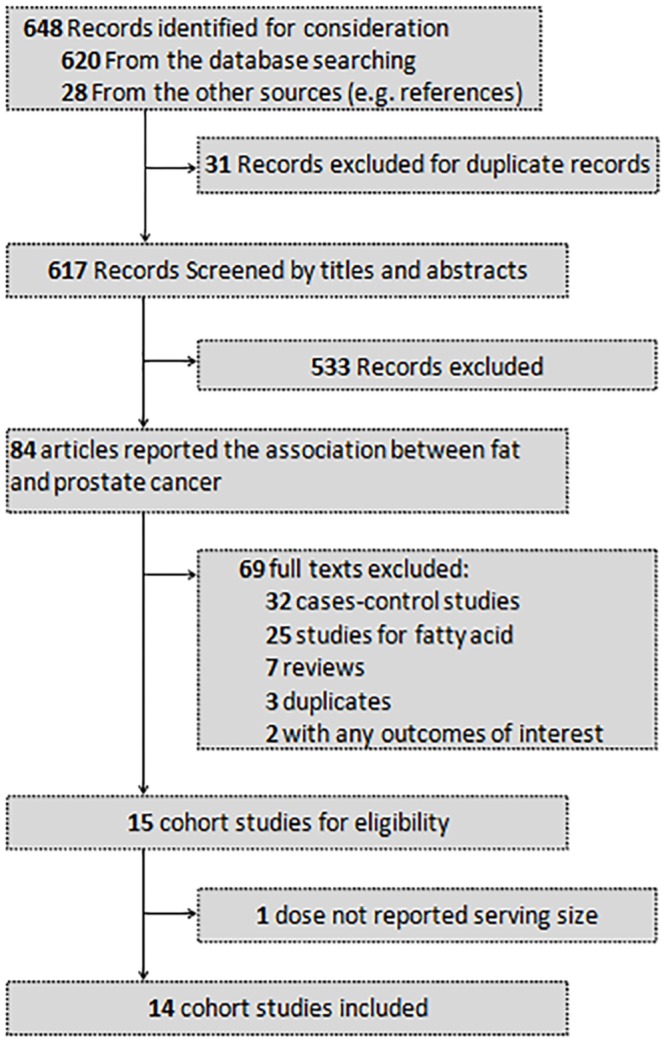
Flow diagram of the literature search.

There were 204 search results in PubMed, 154 in ScienceDirect and 262 in Willey Online Library. After eliminating cohort studies with duplicate and unrelated results, including one study [28] that did not report serving size data, 14 studies [8, 29–41] were included in our meta-analysis with a kappa-value of 0.57 (Fig 1).

### Study characteristics and quality assessment

Among the 14 studies, there were a total of 751,030 participants of which 37,349 cases developed Pca. The mean age of Pca cases was about 60.60 years and the mean follow-up period was 9.2 years. Four studies [29, 30, 33, 37] measured fat intake with energy percentages and the remaining 10 measured grams [8, 32, 34–36, 38–41]. All of the studies were conducted in America or European countries and America (America and Canada) contributed 84.71% of total cases. Twelve studies controlled for the main confounders and two studies [32, 41] adjusted for age only (Tables 1, 2, and 3).

**Table 1:** The main characteristics of included studies.

First author	Year	Age range (years)	Study area	Follow-up	Case/p-years	Type of fat	Main findings (High vs. Low, HRs)		Adjusted variable
							Total Pca	Advanced Pca	
Pelser	2013	From 50 to 71.	Finland	9 years	23,281/288,268	Total fat	—	1.07 (0.95, 1.21)	Age at entry, race, family history of prostate cancer, education, marital status, PSA testing in the past 3 years, physical activity, smoking, self-reported diabetes, BMI at baseline, calories, alcohol, and intake of tomatoes.
						Saturated fat	—	1.21 (1.00, 1.46)	
						Monounsaturated fat	—	0.80 (0.64, 1.01)	
						Polyunsaturated fat	—	1.09 (0.93, 1.28)	
Agalliu	2011	The mean age was 66.2 for cases.	Canada	5 years	661/22,975	Polyunsaturated fat	0.95 (0.70, 1.12)	0.61 (0.34, 1.06)	Age at baseline, race, BMI, exercise activity, education.
Kristal	2010	The mean age was 63.6 for cases.	America	7 years	1,703/9,559	Total fat	—	1.23 (0.58, 2.60)	Adjusted for age, race/ethnicity, treatment arm, and body mass index.
			Canada			Saturated fat	—	0.37 (0.13, 1.00)	
						Monounsaturated fat	—	1.33 (0.41, 4.37)	
						Polyunsaturated fat	—	2.89 (1.24, 6.73)	
Crowe	2008	Mean age were 52.	10 European countries	8.7 years	2,727/142,520	Total fat	0.96 (0.84, 1.09)	—	Adjusted for height, weight, smoking, education, marital status, and energy intake.
						Saturated fat	0.97 (0.85, 1.11)	—	
						Monounsaturated fat	0.98 (0.84, 1.14)	—	
						Polyunsaturated fat	0.98 (0.85, 1.12)	—	

**Table 2:** The main characteristics of included studies.

Park	2007	From 45 to 75.	America	8 years	4,040/82,483	Total fat	0.99 (0.89, 1.09)	0.90 (0.75, 1.09)	Adjusted for time on study, ethnicity, and family history of prostate cancer, education, BMI, smoking status and energy intake.
						Saturated fat	0.94 (0.85, 1.04)	0.87 (0.71, 1.06)	
						Monounsaturated fat	1.01 (0.91, 1.12)	1.03 (0.85, 1.25)	
						Polyunsaturated fat	1.01 (0.91, 1.11)	1.01 (0.84, 1.23)	
Wallström	2007	Mean age were 61.8 for cases.	Sweden	11.0 years	817/10,564	Total fat	0.99 (0.79, 1.24)	1.11 (0.75, 1.66)	Age, diabetes, waist circumference, height, living, educational level, alcohol habits, BMI, smoking history, birth country, total calcium intake, consumption of fruits, vegetables, and red meat.
						Saturated fat	0.98 (0.79, 1.22)	1.08 (0.74, 1.57)	
						Monounsaturated fat	1.01 (0.80, 1.29)	1.22 (0.80, 1.84)	
Neuhouser	2007	From 45 to 69.	America	11 years	890/11,110	Total fat	1.18 (0.84, 1.66)	—	Adjusted for age, race/ethnicity, energy intake, BMI, and smoking. Models with all cases are additionally adjusted for family history.
						Saturated fat	0.97 (0.70, 1.34)	—	
						Monounsaturated fat	1.03 (0.74, 1.44)	—	
Mitrou	2007	From 50 to 69 years old.	Finland	17 years	1267/29,133	Total fat	1.02 (0.85, 1.23)	—	Age, physical activity, Type II diabetes, family history, height, BMI, smoking, cigarettes/day, marital status, energy, education, urban residence.
Hsieh	2003	68.7 for cases.	America	6 years	68/1,665	Total fat	0.72 (0.29, 1.75)	—	Age, the models for protein, carbohydrates, and fat, energy.

**Table 3:** The main characteristics of included studies.

Chan	2000	The mean age was about 57.1.	Finland	8 years	184/27,062	Total fat	1.10 (0.70, 1.70)	—	Alpha-tocopherol, beta-carotene, or both, placebo, education, age, BMI, energy, smoking,
Schuurman	1999	From 55 to 69.	Netherlands	6.3 years	642/9,122	Total fat	1.1 (0.80, 1.52)	—	Age, family history, socioeconomic status, total energy intake.
Veierød	1997	Mean age was 51 for cases.	Norwegian	12.4 years	72/25,708	Total fat	1.30 (0.60, 2.80)	—	Age.
						Saturated fat	0.70 (0.30, 1.50)	—	
						Monounsaturated fat	1.40 (0.60, 3.00)	—	
						Polyunsaturated fat	1.40 (0.60, 3.00)	—	
Giovannucci	1993	From 40 to 75.	America	2 years	279/47,578	Total fat	1.32 (0.91, 1.92)	1.68 (0.97, 2.90)	Energy-adjusted nutrient adjusted for age, calories, body mass index, ancestry, and vasectomy status.
						Saturated fat	0.84 (0.48, 1.47)	0.95 (0.41, 2.21)	
						Monounsaturated fat	1.86 (0.99, 3.51)	1.58 (0.62, 4.00)	
Severson	1989	Not report.	America	17.4 years	174/7,999	Total fat	0.87 (0.58, 1.31)	—	Age-adjusted.
						Saturated fat	1.00 (0.68, 1.46)	—	
						Unsaturated fat	1.09 (0.75, 1.60)	—	

The Newcastle-Ottawa Scale [42, 43], which contains 9 terms with 1 term account for 1 score, was used by two reviewers to assess the quality of the included studies. A third author deal with any disagreements. For each study, we obtained a mean score of 8.07 of all the included studies (Kappa = 0.32) (S1 Table).

### Total fat intake and Pca risk

**Fig 2:**
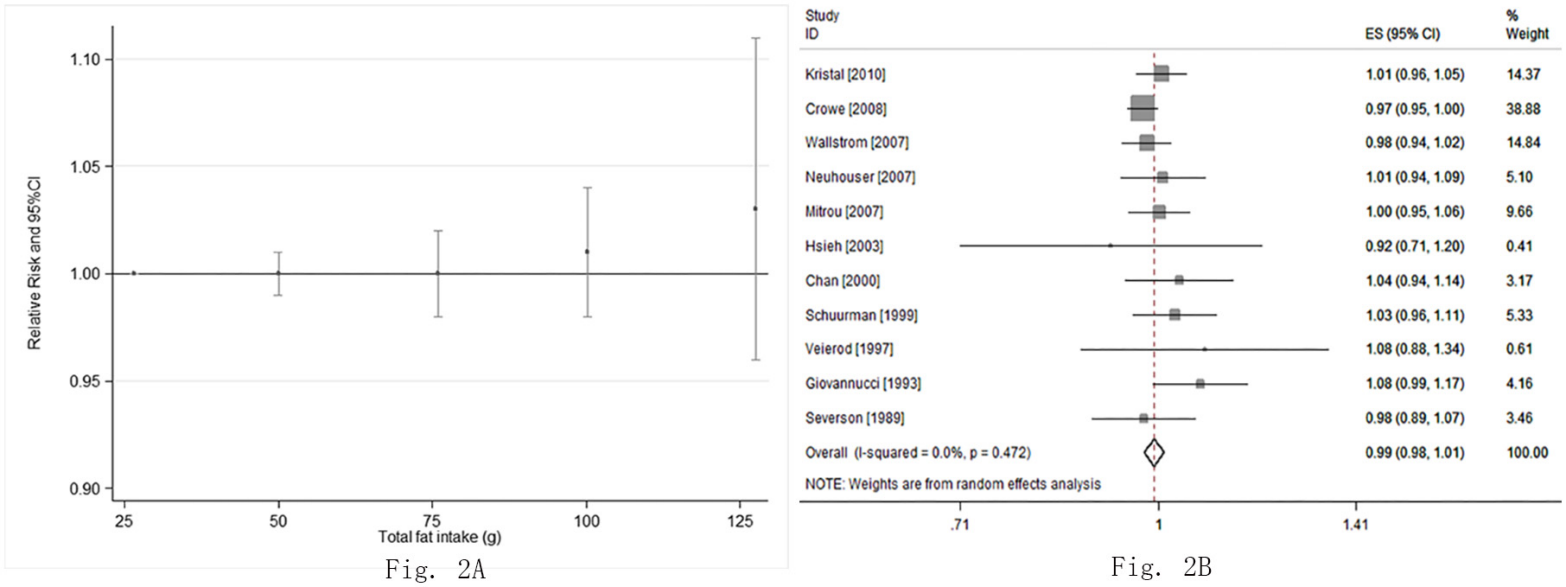
The relationship between total fat intake and risk of Pca. (A) The non-linearity dose-response meta-analysis on total fat intake and risk of Pca. The P value for non-linear test was 0.49. The points assigned to 26.6 g (reference dose), 50 g, 75.95 g, 100.27 g, and 127.6 g, respectively. (B) The linearity dose-response meta-analysis of total fat intake and risk of Pca (every 28.35 g increment a day).

Thirteen studies [29–41] investigated the relevant risk of Pca from total fat intake. We detected no evidence of a non-linear association between total fat intake and the risk for Pca (P = 0.49; Fig 2A). The combined RR was 1.00 (95%CI: 0.99, 1.01; P = 0.94) for every 28.35 g increment of total fat intake a day with, no obvious heterogeneity detected (I^2^ = 5.0%, P = 0.34) (Fig 2A and 2B).

### Saturated fat intake and Pca risk

**Fig 3:**
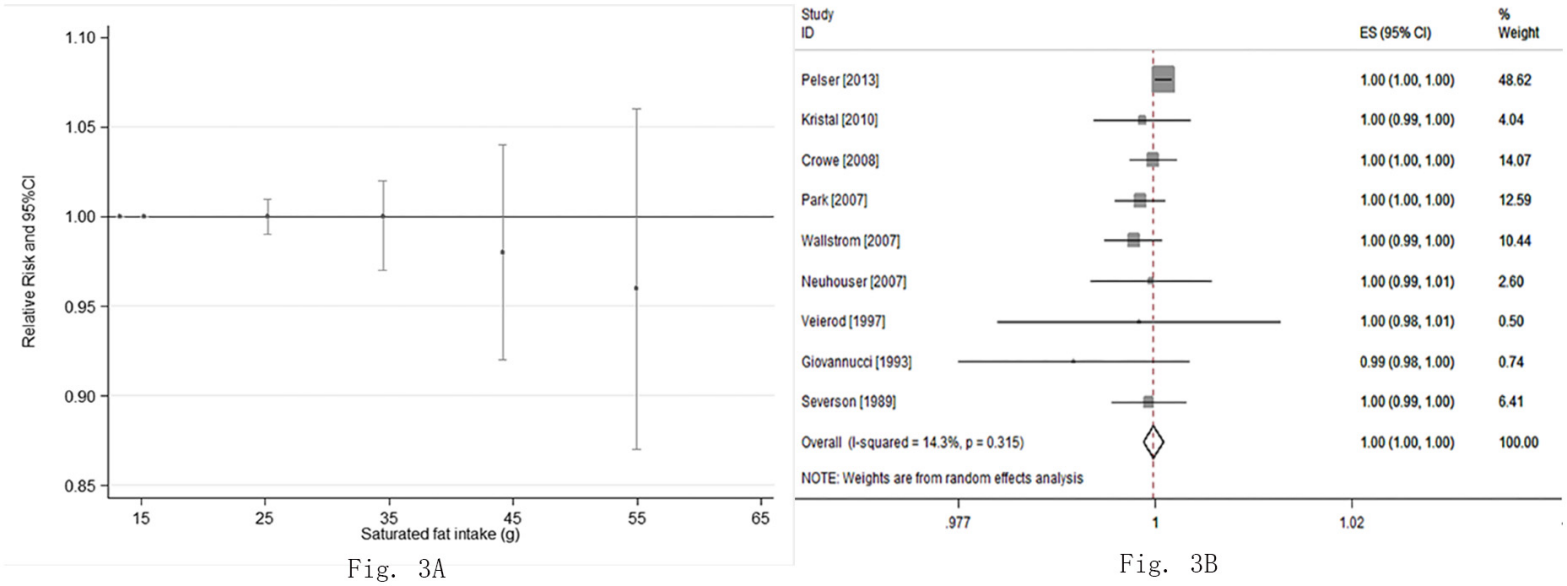
The relationship between saturated fat intake and risk of Pca. (A) The non-linear dose-response meta-analysis on saturated fat intake and risk of Pca. The P value for non-linear test was 0.25. The points assigned to 15.25 g (reference dose), 25.2 g, 34.5 g, 44.16 g, and 54.95 g, respectively. (B) The linearity dose-response meta-analysis of saturated fat intake and risk of Pca (every 28.35 g increment a day).

Nine studies [29–34, 37, 38–41] reported an association between saturated fat intake and risk of Pca. No evidence of a non-linear relationship between saturated fat intake and the risk for Pca (P for non-linearity was 0.25; Fig 3A) was found. The combined RR was 1.00 (95%CI: 1.00, 1.00; P = 0.72; I^2^ = 14.3%) for every 28.35 g (1 ounce) increment of saturated fat consumed per day (Fig 3B).

### Unsaturated fat intake and Pca risk

Ten studies [8, 29–34, 37, 40, 41] reported a risk for Pca due to unsaturated fat intake, seven for polyunsaturated fat [8, 29–33, 37], eight for monounsaturated fat [29–34, 37, 40], and 1 for unsaturated fat [41]. We found no evidence of a non-linear relationship between polyunsaturated fat (P = 0.97) and monounsaturated fat consumption (P = 0.54) and the risk for Pca (S1 and S2 Figs).

**Fig 4:**
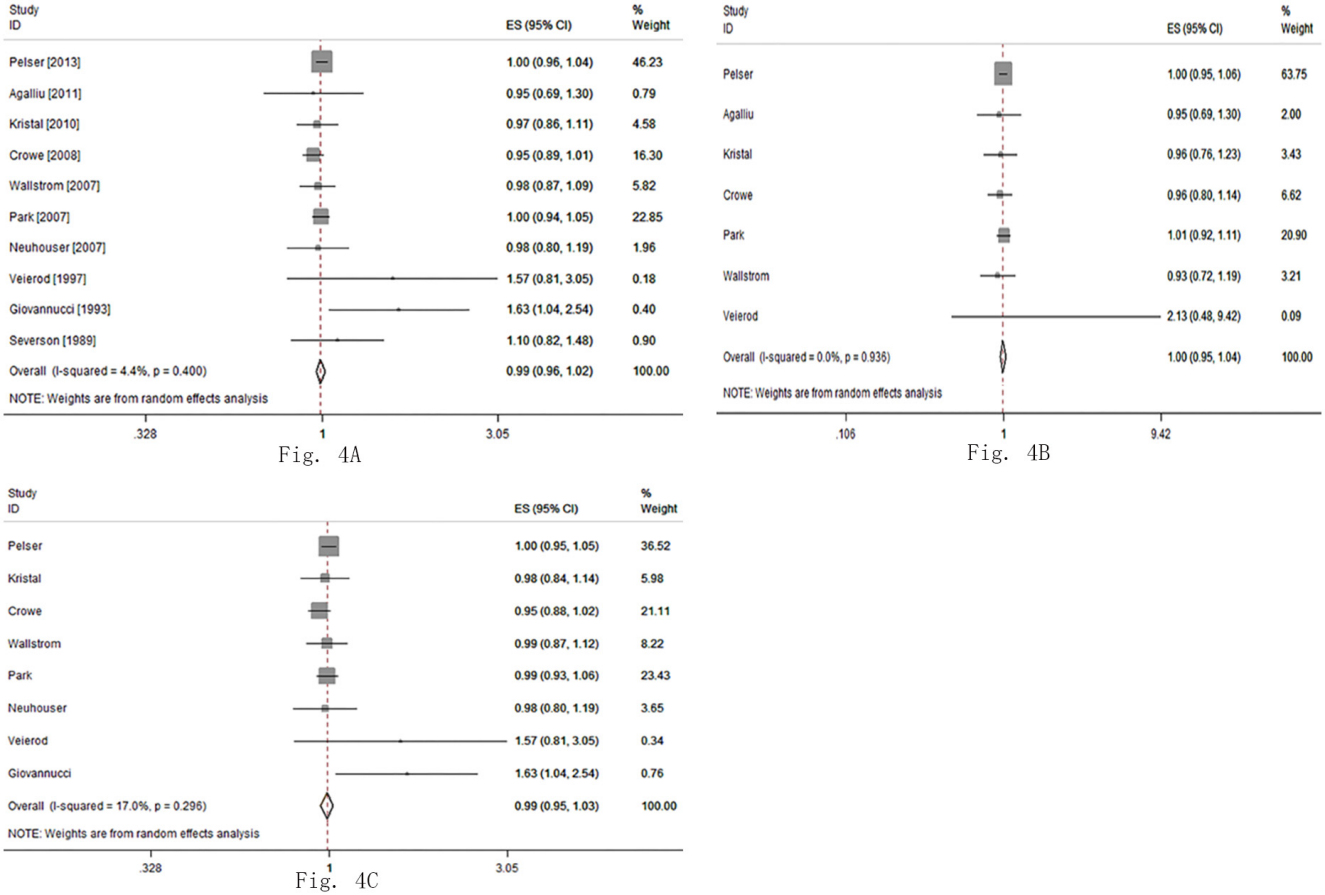
The relationship between unsaturated fat intake and risk of Pca. (A) The linearity dose-response meta-analysis of total unsaturated fat intake and risk of Pca. (B) The linearity dose-response meta-analysis of polyunsaturated fat intake and risk of Pca. (C) The linearity dose-response meta-analysis of monounsaturated fat intake and risk of Pca.

As to a linear association (every 28.35 g increment per day), the combined RR was 0.99 (95%CI: 0.96, 1.02; P = 0.51; I^2^ = 4.4%) for total unsaturated fat intake, 0.99 (95%CI: 0.95, 1.03; P = 0.55; I^2^ = 17.0%) for polyunsaturated fat, and 1.00 (95%CI: 0.95, 1.04; P = 0.85; I^2^ = 0.0%) for monounsaturated fat (Fig 4A–4C).

### Fat intake and the risk for advanced or high grade Pca

**Fig 5:**
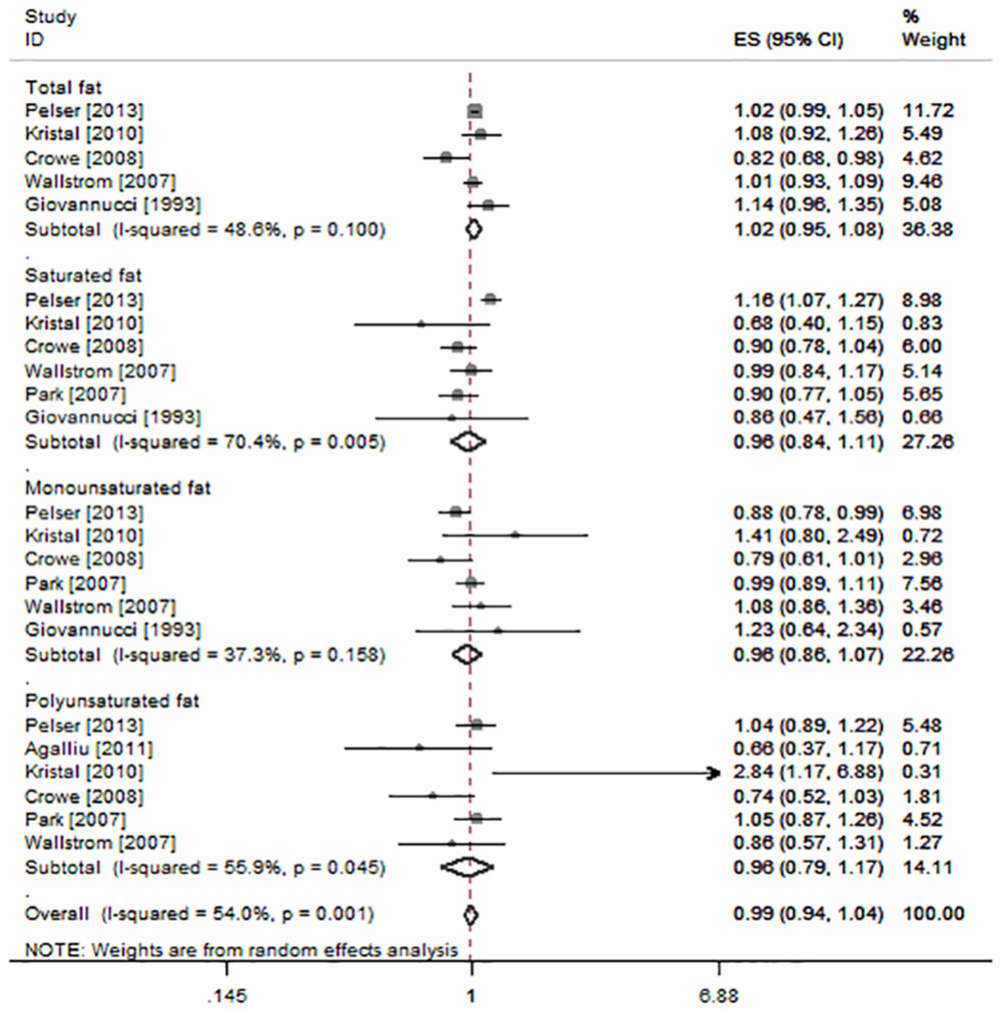
The association between total fat, saturated fat, monounsaturated fat, polyunsaturated fat intake and advanced or high grade Pca (every 28.35 g increment a day). A prostate cancer with a clinical T3a or T3b-T4 N0 or any T or N1, a Gleason score of 8 or higher, or prostate-specific antigen > 20 ng/mL was regarded as high risk of Pca. Some previous studies used a Gleason score of 7 also included here.

Seven studies [8, 29–31, 33, 34, 37] investigated the association between fat intake and the risk for advanced or high grade Pca (Fig 5). The RRs for every 28.35 g/day increment was 1.02 (95%CI: 0.96, 1.08; P = 0.63; I^2^ = 48.6%; n = 5) for total fat, 0.96 (95%CI: 0.84, 1.11; P = 0.61; I^2^ = 70.4%; n = 6) for saturated fat, 0.96 (95%CI: 0.79, 1.17; P = 0.68; I^2^ = 55.9%; n = 6) for polyunsaturated fat, and 0.96 (95%CI: 0.86, 1.07; P = 0.42; I^2^ = 37.3%; n = 6) for monounsaturated fat intake.

### Subgroup, meta-regression, and sensitivity analyses

We conducted a subgroup analysis using primary measurement units (e.g. grams or energy), study area, and adjustment status (adjusted/not) for BMI for potential divergences within subgroups, which showed no substantial change (Table 4). The multivariable meta-regression showed that primary measurement units, study area, adjustment status for BMI were not associated with the risk of Pca. The Knapp–Hartung adjustment *P*-value was 0.32 for total fat, 0.71 for saturated fat, 0.91 for polyunsaturated fat, and 0.65 for monounsaturated fat consumption.

**Table 4:** Subgroup analysis.

Subgroup analysis	Primary measurement unit		Area of country		Adjusted for BMI	
	Energy	Gram	America	European	Adjusted	Non-adjusted
** Total fat (13):**						
Study number	4	9	7	6	8	5
RR* (95%CI)	1.00 (0.99, 1.01)	1.01 (0.98, 1.08)	1.01(1.00, 1.02)	0.99 (0.98, 1.01)	1.01(1.00, 1.01)	0.98 (0.96, 1.00)
*P*-value	0.57	0.62	0.26	0.17	0.29	0.11
Heterogeneity (I^2^)	53.40%	0.00%	0.00%	0.00%	0.00%	0.00%
** Saturated fat (9):**						
Study number	4	5	6	3	6	3
RR* (95%CI)	1.00 (1.00, 1.00)	1.00 (1.00, 1.00)	1.00 (1.00, 1.00)	1.00 (1.00, 1.00)	1.00 (1.00, 1.00)	1.00 (1.00, 1.00)
*P*-value	0.73	0.12	0.98	0.29	0.41	0.76
Heterogeneity (I^2^)	22.10%	0.00%	17.70%	0.00%	43.80%	0.00%
**Total unsaturated fat (10):**						
Study number	4	6	7	3	7	3
RR* (95%CI)	0.99 (0.96, 1.02)	1.04(0.92, 1.18)	1.00 (0.97, 1.03)	0.99(0.96, 1.02)	1.00(0.97, 1.03)	0.99(0.96, 1.02)
*P*-value	0.43	0.52	> 0.99	0.26	0.87	0.86
Heterogeneity (I^2^)	0.00%	28.90%	0.00%	13.60%	0.00%	35.20%
** Monounsaturated fat (8):**						
Study number	4	4	5	3	6	2
RR* (95%CI)	0.99 (0.95, 1.02)	1.09 (0.90, 1.33)	1.00 (0.95, 1.05)	0.97 (0.89, 1.05)	1.00 (0.95, 1.05)	1.07 (0.70, 1.70)
*P*-value	0.37	0.37	0.97	0.41	0.91	0.71
Heterogeneity (I^2^)	0.00%	51.90%	16.70%	18.80%	0.00%	54.30%
** Polyunsaturated fat (7):**						
Study number	4	3	4	3	5	2
RR* (95%CI)	1.00 (0.95, 1.05)	0.95 (0.78, 1.08)	1.00 (0.95, 1.05)	0.95 (0.83, 1.01)	1.00 (0.95, 1.05)	1.00 (0.70, 1.43)
*P*-value	0.95	0.59	0.98	0.51	0.97	0.3
Heterogeneity (I^2^)	0.00%	0.00%	0.00%	0.00%	0.00%	0.00%

*per 28.35 g increment/day.

Sensitivity analysis using the random-effect model showed robust results after omitting one study at a time from each analysis. It also showed consistent results when two studies [30, 33] with most weight in most cases were omitted (S2 Table).

### Publication bias

The Egger’s test did not detect obvious publication bias in our meta-analysis of studies that reported total fat (P = 0.93), monounsaturated fat (P = 0.16), and polyunsaturated fat consumption (P = 0.92). However, obvious asymmetry was observed in saturated fat (P = 0.01). An adjusted meta-analysis using the trim and fill method showed consistent results in both fixed- (RR = 1.00, 95%CI: 1.00, 1.00) and random-effects (RR = 1.00, 95%CI: 1.00, 1.00).

## Discussion

The present meta-analysis confirmed no obvious associations between total, saturated or unsaturated fat consumption and the risk for Pca. Our further analysis also confirmed a lack of association between fat intake and advanced or high grade Pca. Although obvious asymmetry was observed in the Egger’s test of saturated fat intake, the trim and fill method showed consistent results and suggest that asymmetry may be not be caused by publication bias.

Obesity is often linked to high fat consumption [44] while BMI is closely related to Pca [14]. There is a complex relationship between BMI and Pca, but whether BMI increases the risk of Pca remains controversial [45, 46, 47]. For this reason, we conducted a subgroup analysis based upon different statuses of BMI adjustments. Our subgroup analysis showed consistent results in BMI-adjusted and non-adjusted groups, which suggested that our results may not be influenced by BMI. We also conducted a subgroup analysis using primary measurement units and study areas and found no substantial change in the results.

The sensitivity analysis showed robust results in our meta-analysis. Two studies [29, 33] accounted for most of the weight, and in order to determine their potential influence on overall results, both two were omitted in each analysis. The results were consistent and supported the credibility of our meta-analysis.

A previous meta-analysis [12], of 29 observational studies with 5 cohort studies, found that only total fat consumption was associated with an increased risk for Pca (RR = 1.2). Consuming 45g of total fat per day (5 studies, combined RR = 1.12, 95%CI: 1.01, 1.25) or saturated fat (4 studies, combined RR = 1.38, 95%CI: 1.13, 1.70) increased the risk for advanced stage Pca. The meta-analysis was well-designed, but most of the studies included were case-controlled with considerable heterogeneity, which may account for the low grade of evidence. Another systematic review [48] that contained only 5 studies (including one cohort study) claimed that saturated fat consumption was associated with advanced Pca. However, their limited study numbers and sample size may explain the low statistical power of their results. The present meta-analysis is based on large numbers of cohort studies and we found no association between fat intake and the risk for Pca. Our results are similar to a meta-analysis by Chua et al [15]. Our meta-analysis included more high quality cohorts and prepared with more flexible design, may be credible. There were also reviews on this topic [13, 14], but the lack of systematic statistical analysis and less rigorous design may lead to a loss of credibility.

### Potential mechanism of fat intake and Pca risk

There are a few known risks and benefits to consuming dietary fat. Bioactive components in dietary fats such as N-3 polyunsaturated fat acid (n-3 PUFA), may protect against prostate cancer and other types of cancer [49] by altering COX-2 expression and prostaglandin production [50]. Fat-soluble vitamins such as vitamin D and E, are increasingly absorbed as fat is consumed may protect against prostate cancer [51, 52]. In addition to potential carcinogenic pathways being linked to an increase risk Pca, oxidative stress generated during fat metabolism has been reported to increase the risk for Pca [7] though IGF-1 up regulation and increased cell growth [53, 54]. Androgen signaling has been regarded an important factor for Pca progression [14]. Simultaneously, androgen was found to up regulate the IGF-1R expression [55], which may also promote Pca development. Free radicals and proinflammatory fatty acids produced by dietary fat were also considered to promote tumour growth [11]. Since we found no association between fat intake and risk of Pca, the above mentioned factors may generate an offset effect.

### Potential bias

A study by Mills et al [28] of 180 cases and 78,000 person-years was not included in our meta-analysis because it reported a missing serving size in each category (instead of Q1, Q2, Q3, Q4). The RRs in this study were 0.84 (95%CI: 0.52, 1.34), 0.98 (95%CI: 0.59, 1.61), and 1.35 (95%CI: 0.81, 2.23) for Q2, Q3, Q4 compared to Q1 levels of animal fat consumed. This study may bring some bias to our results.

Physical activity status may influence our results since regular physical activity is considered to protect against Pca [56]. Two studies [8, 33] in our meta-analysis controlled for the influence of physical activity on their results. We did not conduct any additional analysis (such as subgroup analysis) for these two studies because the numbers were small and would result in a low statistical power.

Other confounding factors such as age, energy, family history, fruit and vegetable intake, and serum fatty acid levels may have influenced our results. However, sensitivity analysis did not reveal substantial change, which suggests that the influences mentioned above may have little impact on our results.

### Strengths and limitations

To ensure that our results are reliable, we used a dose-response meta-analysis to evaluate high quality cohort studies and to find potential non-linear or linear relationships between fat intake and the risk for Pca. We used subgroup and sensitivity analyses to distinguish the effect among the subgroups and generated consistent results. All of this makes our results more reliable.

There were a few limitations in our meta-analysis as well. First, the limited number of studies included and the considerable amount of heterogeneity detected in our analysis of advanced or high grade Pca and fat consumption may have influenced the accuracy of our results. Second, all studies were conducted in American or European countries. Thus, a selection bias was introduced making this meta-analysis applicable to Americans and Europeans only. Third, we have limit data on addressing the above potential bias in our study that may also influence our results.

## Conclusion

Current published cohort studies suggest no association between total fat, saturated fat, or unsaturated fat intake and the risk for Pca. More studies on the association between fat intake and high grade or advanced stage Pca are needed.

## Supporting Information

S1 Fig The non-linear dose-response meta-analysis on polyunsaturated fat intake and risk of Pca. The P value for non-linear test was 0.97. The points assigned to 4.17 g (reference dose), 10.2 g, 15.31 g, 19.88 g, and 25.47 g, respectively.
(TIF)

S2 Fig The non-linear dose-response meta-analysis on monounsaturated fat intake and risk of Pca. The P value for non-linear test was 0.54. The points assigned to 15.74 g (reference dose), 25.58 g, 35.73 g, and 45.1g, respectively.
(TIF)

S1 PRISMA Checklist The PRISMA Checklist of this meta-analysis. (DOC)

S1 Table Quality access of cohort studies according to Newcastle-Ottawa Scale. (XLSX)

S2 Table Sensitivity analysis results by omitting both the two studies accounted for most the weight. (XLSX)
