# Supplementary figures and images for: Fat Intake Is Not Linked to Prostate Cancer: A Systematic Review and Dose-Response Meta-Analysis
Source: PLoS One. 2015 Jul 17;10(7):e0131747. doi: 10.1371/journal.pone.0131747 (PMC4505895; doi:10.1371/journal.pone.0131747)

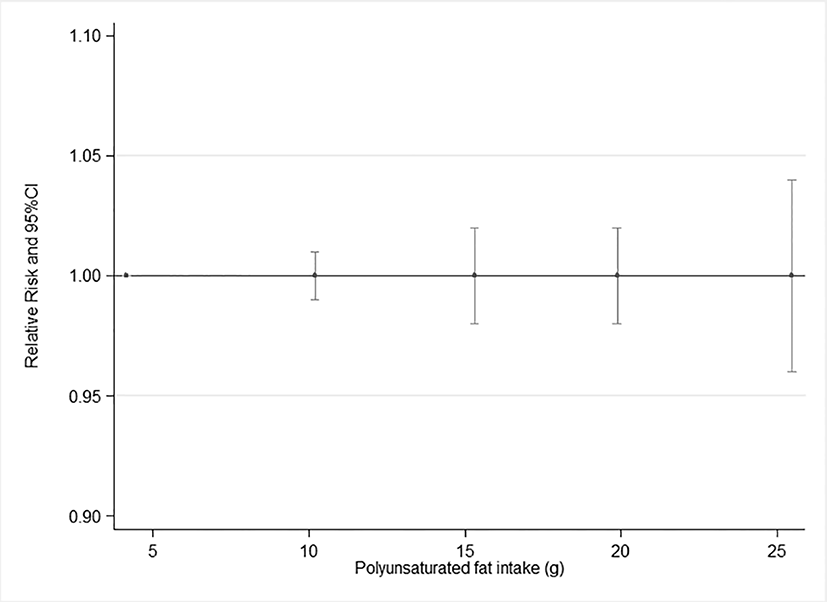

Supplement: S1 Fig — The P value for non-linear test was 0.97. The points assigned to 4.17 g (reference dose), 10.2 g, 15.31 g, 19.88 g, and 25.47 g, respectively. (TIF) [file pone.0131747.s001.tif]

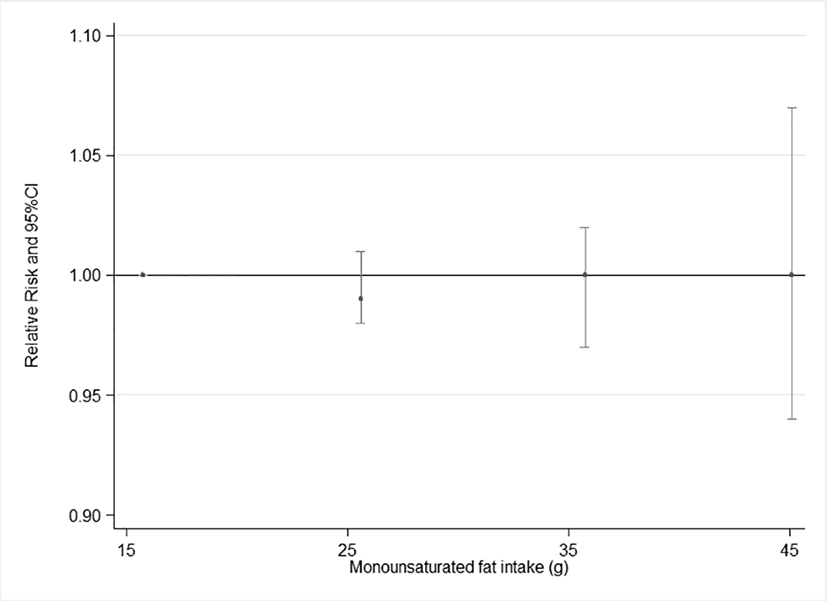

Supplement: S2 Fig — The P value for non-linear test was 0.54. The points assigned to 15.74 g (reference dose), 25.58 g, 35.73 g, and 45.1g, respectively. (TIF) [file pone.0131747.s002.tif]
